# Editorial: Tumor microenvironment complexity and its therapeutic implications

**DOI:** 10.3389/fvets.2025.1543814

**Published:** 2025-01-27

**Authors:** Denner Santos dos Anjos, Marxa Leão Figueiredo, Renee Laufer-Amorim, Carlos Eduardo Fonseca-Alves

**Affiliations:** ^1^Department of Veterinary Surgery and Animal Reproduction, São Paulo State University – UNESP, Botucatu, SP, Brazil; ^2^Department of Basic Medical Sciences, College of Veterinary Medicine, Purdue University, West Lafayette, IN, United States; ^3^Department of Veterinary Clinic, São Paulo State University – UNESP, Botucatu, SP, Brazil; ^4^Institute of Veterinary Oncology - IOVET, São Paulo, Brazil; ^5^Institute of Health Sciences, Paulista University – UNIP, Bauru, SP, Brazil

**Keywords:** canine, immunology, immunotherapy, immuno-oncology, tumor-associated inflammation

## 1 Introduction

The tumor microenvironment (TME) is a dynamic and complex system composed of cancer cells, stromal cells, immune cells, extracellular matrix components, and various signaling molecules (1). This intricate network not only supports tumor growth but also plays a pivotal role in modulating the host immune response. Understanding the interplay between the TME and the immune system is critical for unraveling tumor progression mechanisms and designing more effective therapeutic strategies (2).

Cancer cells within the TME adopt numerous strategies to evade immune surveillance, including the secretion of immunosuppressive cytokines, the recruitment of regulatory immune cells, and the upregulation of immune checkpoint molecules (3). These mechanisms create an immunosuppressive environment that hinders the anti-tumor immune response, enabling the tumor to grow unchecked. Additionally, the role of stromal components, such as cancer-associated fibroblasts (CAFs) and the extracellular matrix (ECM), further complicate this scenario by providing physical barriers to immune cell infiltration and by influencing cellular signaling pathways (4, 5).

The immune system, on the other hand, demonstrates a dual role within the TME, acting both as a tumor suppressor and a promoter (5). While cytotoxic T lymphocytes (CTLs) and natural killer (NK) cells are essential for targeting and eliminating cancer cells, other immune subsets, such as myeloid-derived suppressor cells (MDSCs) and tumor-associated macrophages (TAMs) can facilitate tumor progression through their immunosuppressive and pro-angiogenic activities (4). Recent advancements in immunology and oncology have led to a deeper understanding of these interactions, paving the way for the development of innovative therapies, such as immune checkpoint inhibitors, cancer vaccines, and adoptive cell therapies. These approaches aim to reprogram the TME to restore immune surveillance and enhance anti-tumor immunity (1).

This editorial brings an overview of the papers published in our Research Topic and expands our knowledge regarding the multifaceted interactions between the TME and the immune system. It highlights key mechanisms of immune evasion and discusses the latest therapeutic advancements targeting these pathways. By elucidating these complex relationships, we aim to contribute to the ongoing efforts in developing more effective and durable cancer treatments. Six articles were selected for this Research Topic, showcasing current advances in TME research. Below, we provide a summary of the main findings of the article and invite readers to explore the full manuscripts.

Kim et al. investigated in an open-source canine scRNA-seq dataset how triple-negative canine breast cancer (TNBC) induces immune suppression in dogs. Their study presented potential mechanisms through which TNBC modulates the immune-suppressive tumor immune microenvironment (TiME). An enrichment of immune suppressive gene sets was observed in TNBC related to TGF-β, TNF-α, anergy, anti-inflammatory responses, M2 macrophages, and T cell exhaustion. Another important result was the upregulation of genes associated with immune suppression, such as SPP1, HMGA1, and WNT5A. In addition, interactome analysis identified significant interactions between distinct subsets of cancer cells and other cells, such as CD4+ and CD8+ T cells, mediated through paracrine and autocrine communication. Finally, the expression of TBNC ligand and receptor genes in immune-modulatory cancer cell subpopulations suggested a role in establishing an immune suppressive environment through cancer-T cell and cancer-cancer cell interactions. In conclusion, understanding this TiME may inform us of the important role of the immune system, serving as a potential target for anticancer immunotherapy.

Considering these interactions between cancer-T cells, Diehl and Hansmann investigated possible immune checkpoint molecules and interferon-mediated immune response factors involved in the regression of canine cutaneous histiocytoma (CCH). They evaluated 48 young dogs (≤ 4 years of age) diagnosed with CCH, dividing them into four groups (Groups 1–4) based on the progression of tumor cells in parallel with an increase in infiltrating lymphocytes. Immune checkpoint expressions of CD80, PD-L1, and CD86 were observed in all neoplastic samples, with a variable degree of staining intensity. The density for CD80 immunolabeled tumor cells showed a significant decline from Group 1 to Groups 3 and 4. This is interesting since these immune checkpoints may be involved in the induction of immunotolerance, suggesting that a decline in CD80 might be related to the activation of T-cell mediated anti-tumor immune responses. In this Research Topic, two novel findings are worth mentioning. First, the constitutive expression of CD86 and CD80 in regressing Langerhans cells (LC) lesions in canines represents a novel finding that could inform future anti-tumor strategies. Second, the expression of mx1, a transcription factor triggered by type-I-interferons, was identified in CCH for the first time, opening intriguing questions about its role in cancer biology. Finally, when evaluating Survivin, a protein linked to tumor survival, half of the samples in all groups showed more than 5% immunolabeled tumor cells. Interestingly, a moderate negative correlation between the mitotic count and cleaved caspase-3 positive cells was observed, showing a higher rate of apoptosis in the regression phase compared to earlier phases, suggesting a plausible contribution to tumor regression.

Garcia A. P. V. et al. discussed collagen modifications in canine mammary tumors and their potential role in predicting lymph node metastasis, since collagen changes may be a significant protein in tumor invasiveness. In canine carcinoma mixed tumors with metastasis, the shorter and wavier collagen fibers observed compared to carcinoma without metastasis may be associated with tumor aggressiveness and progression. A plausible explanation could be the breakdown of collagen fibers by metalloproteinases, which in turn could contribute to the invasion of neoplastic cells, facilitating lymphatic invasion.

Lin et al. evaluated the potential inhibitory effect of sodium butyrate (NaB) on the replication of Marek's disease virus (MDV) and lymphoma formation. Surprisingly, the authors demonstrated the capacity of NaB to inhibit the expression of MDV early genes, such as ICP4 and ICP27, showing decreased tumorigenesis in the chicken spleen. This is important since chickens infected with a virulent strain of MDV develop aggressive T-cell lymphomas. Taking this into account, the authors also observed that NaB upregulated tumor-suppressor genes, such as PTEN, TGF-β, and ARRDC3, showing a remarkable ability to reduce the morbidity of lymphoma in chickens. Another important finding was the ability of NaB to induce apoptosis in lymphoma cells by enhancing the expression of molecules involved in the mitochondrial signaling pathway, overall contributing to apoptosis. This highlights the potential inclusion of NaB into lymphoma prevention regimens and warrants future studies for its use in viral diseases.

Another important study we highlight here was by Altamura and Borzacchiello, which explored the capacity for the anti-EGFR monoclonal antibody Cetuximab to control feline oral squamous cell carcinoma (OSCC) signaling. They reported a decrease in the downstream effector pAKT as well as pEGFR levels across all cell lines (SCCF1, SCCF2, and SCCF3). These findings are similar to those from human head and neck SCC (HNSCC), in which anti-EGFR monoclonal antibodies have been used in clinical practice, since around 90% of HNSCC cases show over-expression of EGFR, suggesting a more aggressive disease. Furthermore, the use of Cetuximab induced apoptosis associated with PARP cleavage and BAX accumulation, promoting cell death in all cell lines. These results are lapidary and start to close our gap for new therapies for this lethal cancer in cats.

Finally, Garcia J. S. et al. evaluated inflammatory indices before giving immunotherapy alone or in combination with metronomic chemotherapy. Among the inflammatory indices analyzed, the C–reactive–protein–albumin ratio above the cut-off of 1.9 had the worst progression-free survival (PFS) and overall survival (OS) rates. In addition, blood-circulating Treg lymphocytes were correlated with worse PFS and OS rates, suggesting dysfunction or loss of T cell function in the tumor microenvironment, and characterizing an immunosuppressive profile.

In summary, the articles included in this Research Topic summarize and discuss the main results related to the TME, spotlight novel potential targeted therapies, and identify potential immune checkpoint molecules, cancer-cancer, and cancer-T cell interactions, providing a more personalized profile that we envision can guarantee, in the future, more individualized treatment regimens.
